# Death in the Well

**Published:** 1871-08

**Authors:** 


### DEATH IN THE WELL.

ALWAYS before going into a vault, or well, or deep pit, let down a light; if it goes out, death is at the bottom, because there is carbonic acid gas there, which is an air that has no support for the lungs, gives no life to the blood, and we as certainly die, and as soon, as if we had no air at all,—as if smothered, or in the water, or hung. But if a person has fallen into the well and some one must go down, the quickest plan to purify the air, and to get rid of the carbonic acid gas, is to throw quick-lime into the well, if there is any water at the bottom; if not, throw some in; this throws out heat, which rarefies the carbonic acid and sends it to the top; besides, the dissolving lime absorbs it.

Large quantities of cold water thrown in is a substitute; or set shavings or paper on fire and let them fall to the bottom; the fresh water absorbs, the flame rarefies the gases.

